# Influenza A in Wild Boars: Viral Circulation in the Emilia-Romagna Region (Northern Italy) between 2017 and 2022

**DOI:** 10.3390/ani12121593

**Published:** 2022-06-20

**Authors:** Alice Prosperi, Laura Soliani, Elena Canelli, Laura Baioni, Valentina Gabbi, Camilla Torreggiani, Roberta Manfredi, Irene Calanchi, Giovanni Pupillo, Filippo Barsi, Patrizia Bassi, Laura Fiorentini, Matteo Frasnelli, Maria Cristina Fontana, Andrea Luppi, Chiara Chiapponi

**Affiliations:** 1 OIE Reference Laboratory for Swine Influenza, Istituto Zooprofilattico Sperimentale della Lombardia e dell’Emilia Romagna (IZSLER), 25124 Brescia, Italy; laura.soliani@izsler.it (L.S.); laura.baioni@izsler.it (L.B.); valentina.gabbi@izsler.it (V.G.); camilla.torreggiani@izsler.it (C.T.); roberta.manfredi@izsler.it (R.M.); irene.calanchi@izsler.it (I.C.); giovanni.pupillo@izsler.it (G.P.); filippo.barsi@izsler.it (F.B.); patrizia.bassi@izsler.it (P.B.); laura.fiorentini@izsler.it (L.F.); matteo.frasnelli@izsler.it (M.F.); mariacristina.fontana@izsler.it (M.C.F.); andrea.luppi@izsler.it (A.L.); chiara.chiapponi@izsler.it (C.C.); 2 Swine Pratictioner—ECPHM Diplomate, 42030 Viano, Italy; elena.canelli@yahoo.it

**Keywords:** influenza A virus, wild boar, viral circulation, genetic characterization

## Abstract

**Simple Summary:**

Wild boars and feral pigs are underinvestigated hosts for influenza A viruses (IAVs). This study confirmed and evaluated viral circulation in the Emilia-Romagna wild boar population between 2017 and 2022. Samples were collected at post mortems and screened for IAVs; 0.37% of the tested animals provided positive results. Positive samples were subtyped, isolated, and genotyped via full-genome sequencing. The results highlight the co-circulation of the same viral genotypes in overlapping years in both pigs and wild boars in the same geographical area. Considering the role of domestic and wild *Sus scrofa* species in the IAVs’ ecology, surveillance against these viruses in the wild boar population needs to be implemented.

**Abstract:**

A systematic surveillance against influenza A viruses (IAVs) in the *Suidae* population is essential, considering their role as IAV mixing vessels. However, the viral circulation in wild *Sus scrofa* species is poorly investigated in comparison to the knowledge of IAV infection dynamics in domestic pigs. This study investigated the circulation and the genetic diversity of wild boars’ IAVs detected in the Emilia-Romagna region (2017–2022). A total of 4605 lung samples were screened via an M gene real-time RT-PCR for SwIAV; positive samples were subtyped by multiplex RT-PCR, and viral isolation was attempted. Isolated strains (3 out of the 17 positives) were fully sequenced to evaluate viral genotypic diversity. H1N1 was the most frequently detected subtype, with identification of H1pdm09N1 and H1avN1. Whole-genome phylogenetic analysis revealed SwIAVs belonging to different genotypes, with different genetic combinations, and highlighted the simultaneous circulation of the same genotypes in both pigs and wild boars, supporting the hypothesis of SwIAV spillover events at the wildlife–livestock interface. This study represents an update on the wild boar SwIAV Italian situation, and the strains’ complete genome analysis showed an evolving and interesting situation that deserves further investigation.

## 1. Introduction

Wildlife viruses can easily spill over into human and domestic animal populations, representing an underinvestigated burden for both public and veterinary health [1,2]. Influenza viruses (IVs), belonging to the genus *Influenzavirus A* (IAV) within the *Orthomyxoviridae* family, are the most widespread agents of respiratory infections in humans and swine (*Suidae*) [3,4]. Moreover, both of them have a relatively similar pattern of respiratory receptors (a preponderance of sialic acid α2,6-galactose-linked receptors and a low number of α2,3), which is determinant in the host range establishment and explains the comparatively frequent human virus incursions into the swine population [5,6]. IAVs are negative-sense single-stranded RNA viruses and their genome is divided into eight segments (PB2, PB1, PA, HA, NA, NP, NA, M and NS) [7,8]. Therefore, both the genome structure and the wide host range allow for the viral hypervariability and the creation of new viral variants, which might lead to pandemic events, as happened in 2009 (H1N1pdm09) [9,10]. IAVs are classified into subtypes based on the hemagglutinin (HA) and neuraminidase (NA) glycoproteins’ genetic and antigenic structures [11,12,13]. The swine influenza viruses (SwIAVs) circulating in Europe belong mainly to four IAV lineages: (i) the “avian-like swine H1N1” (H1avN1; clade HA-1C) lineage; (ii) the “human-like reassortant swine H3N2” (H3N2) lineage; (iii) the “human-like reassortant swine H1N2” (H1huN2; clade HA-1B) lineage; and (iv) the “pandemic-like swine H1N1” (H1N1pdm; clade HA-1A.3.3.2) lineage [13,14,15].

IAV infection in a pig population usually leads to a highly contagious respiratory disease, which is mainly characterized by high morbidity (up to 100%) and low mortality rates [16,17,18]. Although the knowledge of IAV infection dynamics in pig hosts is constantly evolving worldwide, viral circulation in wild boars and feral pigs is poorly investigated, despite the fact that the three of them belong to the *Sus scrofa* species [19], and only a few reports are available about the Italian situation [20,21]. European epidemiological surveys on IAV circulation in the Eurasian wild boar population demonstrated a seroprevalence ranging between 1.4% and 25.9% [3,19,20,21,22,23,24,25,26] and a virological prevalence between 0.8% and 3.4% [3,20,21]. In addition, the wild boar ecological niche enables the possibility of IAV spillover events from wild birds to wild *Sus scrofa* species [19], as has been reported with serological evidence in the US feral swine population [27].

Wild boars have dramatically increased in number throughout Italy in recent years, resulting in significant agricultural damage and representing a burden for viral infectious disease spread [28]. The latest virological Italian data, published in 2012, reported an H1avN1 subtype in the wild boar population of the Emilia-Romagna region which was a predominant strain co-circulating in the domestic swine population in the same period [20].

The main aim of this study was to investigate the current IAV viral circulation in the Emilia-Romagna wild boar population, taking into account the increased genetic variability of the circulating strains among the domestic swine population [29]. For this purpose, the identified IAVs were subtyped and genotyped, and the evaluation of the results in a high-density commercial swine production geographical area was performed.

## 2. Materials and Methods

### 2.1. Study Design and Sampling

Virological surveillance was performed in the Emilia-Romagna region (Northern Italy) between 2017 and 2022 via an active and passive surveillance program, using the samples collected during the Regional Wildlife Control Plan. Sampling was organized with the collaboration of the Regional Agriculture Office, Fishing and Hunting Offices, and local veterinary services; the surveillance was carried out in the whole regional territory, covering 8 out of the 9 provinces of the region (Bologna, Forlì-Cesena, Modena, Parma, Piacenza, Ravenna, Reggio Emilia, Rimini).

Wild boars’ carcasses or viscera were collected and delivered, refrigerated, to the IZSLER laboratories by rangers, hunters, and local veterinary authorities.

### 2.2. SwIAVs Detection, Subtyping and Viral Isolation

Lung specimens conferred to the IZSLER laboratories were homogenized in 1:10 in phosphate-buffered saline (PBS); total RNA was extracted from 100 µL of the homogenized sample according to the One for All vet kit (Indical Bioscience GmbH, Leipzig, Germany) manufacturer’s instruction.

Samples were firstly screened using an M gene TaqMan one-step real-time RT-PCR for influenza A virus, according to Slomka and colleagues [30], and viral RNA was amplified with influenza-specific primers and probes (M+24 F primer 5′-AGA TGA GTC TTC TAA CCG AGG TCG-3′, M−124 R primer 5′-TGC AAA AAC ATC TTC AAG TCT CTG-3′, M-124 Rev-mod: 5′-TGC AAA GAC ACT TTC CAG TCT CTG-3′; M+64 probe 5′-FAM-TCA GGC CCC CTC AAA GCC GA-BHQ1-3′) [30].

All of the positive samples were identified at the subtype level using a nested multiplex end-point RT-PCR, as previously described [29], which allowed viral differentiation into the subtypes H1avN1, H1huN2, H3N2, and H1pdm09N1 according to the amplicon product length (for the HA gene the primers set: H1avN1 for 5′-CTG CAC TGA AAG CTG ACA CC-3′ and H1avN1 rev 5′-GCT GCT CCC TTA ATT CCT CA-3′, H1huN2_for 5′-GCT ACC ATG CGA ACA ATT CA-3′ and H1huN2_rev 5′-TCA GCA TTT GGT GTT TCT GC-3′, H1pdm09N1 for 5′-CAG ACA CTG TAG ACA CAG TAC-3′ and H1pdm09N1 rev 5′-CTA GTA GAT GGA TGG TGA ATG C-3′, H3_for 5′-CAR ATT GAR GTG ACH AAT GC-3′ and H3_rev 5′-GGT GCA TCT GAY CTC ATT A-3′; For the NA gene the primers set: N1_for 5′-TGA AAT ACA ATG GCA TAA TAA C-3′ and N1_rev 5′-GGA TCC CAA ATC ATC TCA AA-3′, N2_for 5′-GGA AAA GCA TGG CTG CAT-3′ and N2_rev 5′-GTG CCA CAA AAC ACA ACA AT-3′) [29].

Simultaneously, viral isolation from positive samples was attempted, inoculating both 11-day-old SPF embryonated chicken eggs and susceptible cell lines (MDCK and Caco-2) [17,31].

### 2.3. Genetic Characterization of the Isolated Viruses

SwIAVs isolates were further genetically and antigenically analyzed using next-generation sequencing (NGS) with Illumina technology (MiSeq Sequencing System-Illumina Inc., San Diego, CA, USA), as recently reported [29]. Gene sequences of the eight IAV genome segments, such as gene combinations, were compared with SwIAVs circulating in domestic pigs in the same geographical area [29] and with sequences retrieved from the GenBank Influenza virus resource database (https://www.ncbi.nlm.nih.gov/genomes/FLU/Database/nph-select.cgi?go=database; accessed on 26 April 2022). The sequences of SwIAV HA gene segments were then aligned with ClustalW using MEGAX [32], and maximum-likelihood (ML) phylogenetic tree analysis was performed using the IQ-TREE-2 software [33,34,35]. The phylogenetic tree robustness was statically evaluated by bootstrap analysis with 1000 bootstrap replicates. The phylogenetic tree was visualized using Figtree v. 1.4.4 (http://tree.bio.ed.ac.uk/software/figtree, accessed on 26 April 2022), and the origin of each segment was determined by its clustering with reference strains (Supplementary S1-dataset of sequences used in this study). Finally, wild boar H1 sequences were analyzed and named using the swine H1 influenza classification tool (http://www.fludb.org, accessed on 26 April 2022) [14].

## 3. Results

### 3.1. SwIAV Subtypes Circulation (Years 2017–2022)

**Figure 1:**
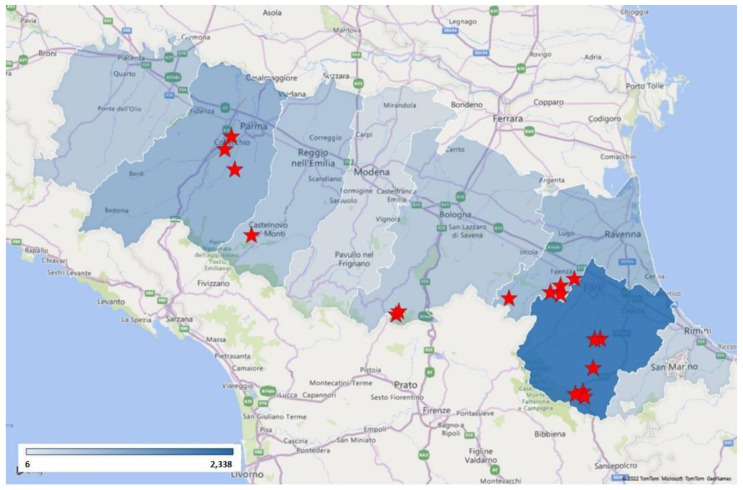
Sampling abundance evaluated for province distribution. The map shows sampling concentration (expressed with the blue intensity) and PCR-positive wild boars (red stars).

From 1 January 2017 to 4 April 2022, 4605 wild boar lung samples were analyzed in 2414 SwIAV one-step real-time RT-PCR tests. The lungs were pooled according to date and location of sampling, with a maximum of five individuals per pool. The sampling abundance evaluated for province distribution is reported in Figure 1.

During the study period, 17 of the examined samples (0.37%) tested positive in the M gene one-step real-time RT-PCR. Of them, seven (41.18%) SwIAVs were subtyped using the nested multiplex end-point RT-PCR; the low percentage of subtyped samples depends mainly on the weak positivity (high Ct value) in the M gene RT-PCRs or on the carcasses’ conservation.

Out of the subtyped wild boars SwIAVs, three belonged to the H1pdm09N1 subtype (clade HA-1A; 43%), two belonged to the H1avN1 subtype (clade HA-1C; 29%), one belonged to the H1avNx subtype (clade HA-1C, N not-typed; 14%), and one belonged to the H1N2 subtype (clade HA-1B; 14%) (Table 1).

### 3.2. SwIAV Genotypes and Phylogenetic Analysis

Three out of the seventeen (17.65%) positive samples were isolated and fully genetically characterized via an NGS approach. The isolated strains were from wild boar samples collected in different years and from disparate geographical areas: (i) a 2018 strain from Ravenna province (GenBank accession number MW136322-MW136329); (ii) a 2020 strain from Parma province (GenBank accession number MW621858-MW621865); and (iii) a 2021 strain from Forlì-Cesena province (GenBank accession number ON468439-ON468446).

Whole-genome phylogenetic analysis of the isolates was performed, and the eight genome segments were aligned and analyzed in order to assign each virus to a specific lineage, genotype, and origin. Two of them were subtyped as H1N1 but belonged to different genotypes: the 2018 isolate was an HA-1C-N1av (H Clade 1C.2.1) in genotype U, while the 2020 isolate was an HA-1C-N1av (H Clade 1C.2.1) in genotype A (Harder’s nomenclature [36]). Lastly, the 2020 isolate was an H1pdm09N1-derived subtype (HA-1A-N1av—Clade 1A.3.3.2) in genotype 31 (Table 2). Those results were also compared with the SwIAVs circulating in domestic pig hosts and are reported in Table 2.

**Figure 2:**
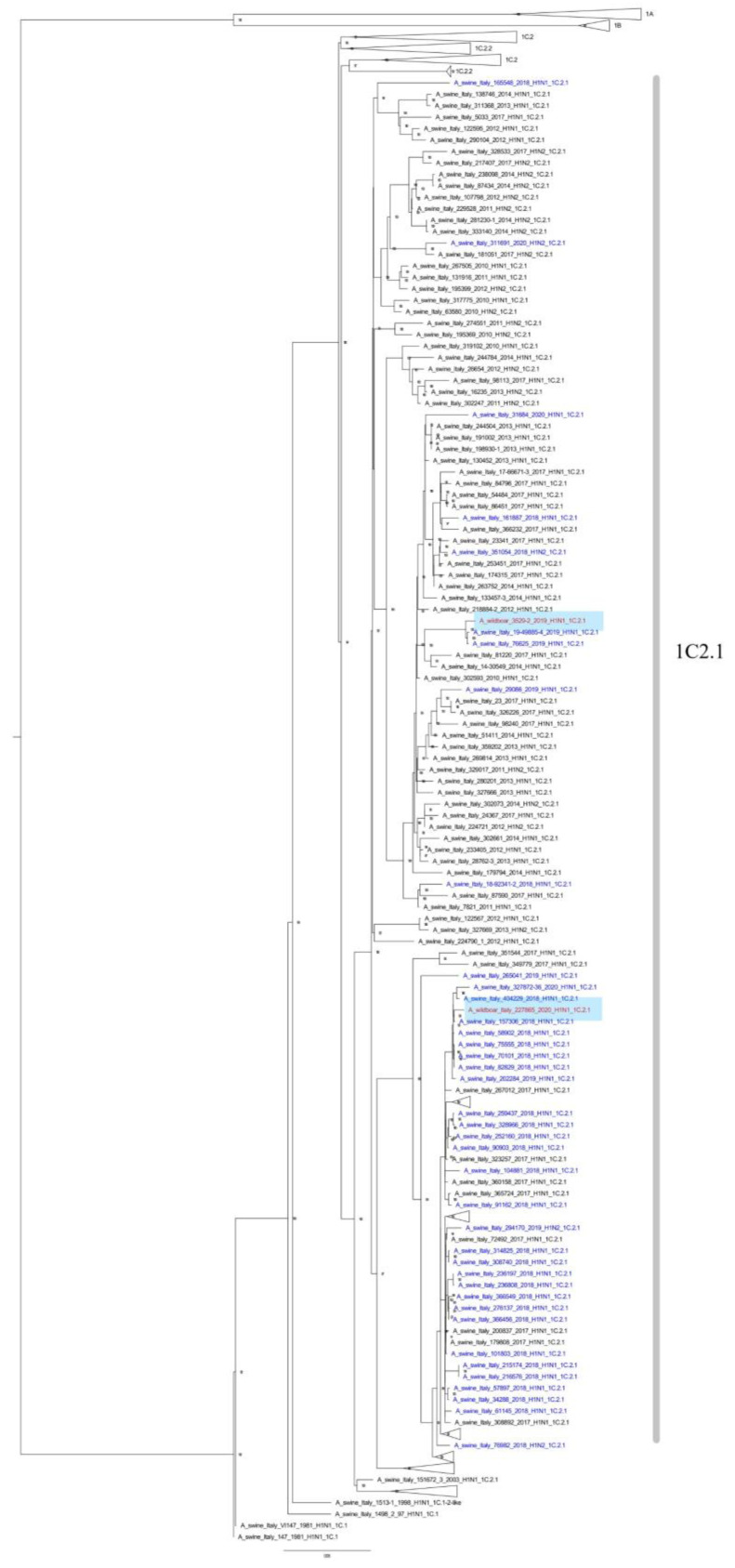
ML phylogenetic tree showing the wild boar A/wild_boar/Italy/3529/2019 and A/wild_boar/Italy/227865/2020 swIAVs detected in this work together with 316 HA-H1C Italian sequences (1575 nucleotides) of strains recently analyzed. The tree was inferred with 507 recently-described Italian sequences (ref. [29,38]) retrieved from GenBank and collected in the years 2009–2020. The detected wild boar swIAVs are written in red. Strains detected since 2018 are in blue, older strains are in black. Bootstrap values under 70% are not shown, clades 1A, 1B, 1C.2, and 1C.2.2 have been collapsed; scale bar represents the number of nucleotide substitutions per nucleotide site.

**Figure 3:**
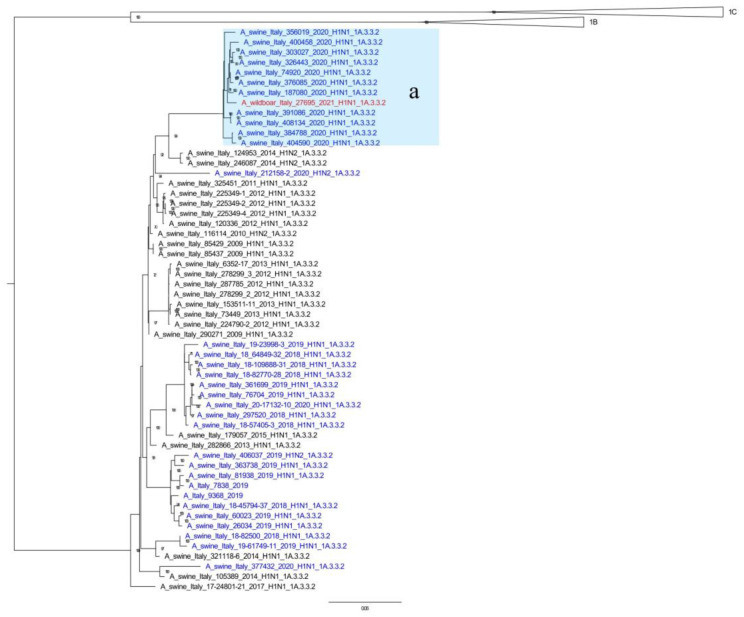
ML phylogenetic tree showing the wild boar A/wild_boar/27695/2021 swIAV detected in this work together with 55 HA-H1A (1A.3.3.2) Italian sequences (1575 nucleotides) of strains recently analyzed. The tree was inferred with 507 recently-described Italian sequences [29,38] retrieved from GenBank and collected in the years 2009–2020. The recently-described [29] sub-cluster *a* was highlighted in pale blue. The detected wild boar swIAV is written in red. Strains detected since 2018 are in blue, and older strains are in black. Bootstrap values under 70% are not shown; scale bar represents number of nucleotide substitutions per nucleotide site.

Furthermore, phylogenetic analyses of the HA gene showed that the wild boar SwIAV isolates obtained in this study were closely related to those identified in the domestic pig population. The 2019/3529 strain (HA Clade 1C.2.1), sampled at the end of 2018, was closely related (range of nucleotide identity 99.01–99.74 for all gene segments) to a SwIAV detected in 2019 in the Veneto Region (A/swine/Italy/76625/2019). The 2020 isolate clustered with the most prevalent swine Italian clade (HA Clade 1C.2.1) [29] (Figure 2). Finally, the 2021 isolate fell within the sub-cluster *a*, and it had a new genotype which was recently isolated [29] as a new antigenic variant circulating in the domestic pig population in the same geographical area (Figure 3).

## 4. Discussion

Swine have a well-known crucial role in IAVs’ ecology and in viral interspecific transmission, being susceptible to both avian and human IVs and thus acting as an IAV “mixing vessel”. This capability leads to the development of multiple reassortant strains (avian/human, human/swine, or human/avian/swine reassortants), which are agents of spillover infections and are crucial in viral epidemiology [4,6,39,40]. Even though wild boars and feral pigs both belong to the *Sus scrofa* species and can be infected with both avian and swine IAVs in the same way as domestic pigs [19,27], these hosts and their role in the SwIAVs’ ecology are underinvestigated. To date, several reports about SwIAV seroprevalence in European countries are available [3,19,20,21,24,25,26,41,42], but only a few of them are about virological circulation [19,20,21] and the genotyping of the viral isolates [20].

During this study, a total of 4605 wild boar biological samples were investigated for SwIAVs to evaluate the active viral circulation; of these, 17 tested positive (0.37%). The relatively low prevalence detected could have two explanations: a comparatively low endemic circulation in the wild boar hosts or a not completely representative sampling. The positivity level observed in this study could support the evidence of the occurrence of epidemic outbreaks in the wild hosts. At the same time, convenience sampling performed during the Regional Wildlife Control Plan might not be truly representative of the viral circulation in the wild boar population. To support this hypothesis, further investigations are certainly needed, such as the use of different samples or sampling procedures; for instance, Vittecoq et al. [19] reported a 1% SwIAV prevalence (95% confidence) in wild boars’ nasal swabs collected in the Camargue area.

At the subtype level, H1N1 was the most frequently detected subtype (five out of seven), identified as H1pdm09N1 (three samples) and H1avN1 (two samples). The identification of the subtype using the nested multiplex end-point RT-PCR is strongly recommended for isolated viruses; however, viral isolation was successful in 3 samples out of the 17 positives. To the authors’ knowledge, the explanation of these results is due to the weak positivity (high Ct value) in the M gene RT-PCRs or the carcasses’ conservation.

The NGS analysis of the isolated strains showed an evolving and interesting situation that deserves further investigation. The 2018 strain (genotype U) was a rare genotype detected in Italy (1.1%) but was closely related (nucleotide identity 99.01–99.74 for all gene segments) to a SwIAV reported in a swine farm in 2019 in the Veneto region (A/swine/Italy/76625/2019) (Figure 2); these were the only genotype U reports in Northern Italy and were from non-related geographical areas.

The HA-1C.2.1-N1av strain isolated in 2020 belonged to SwIAV genotype A, which is the dominant H1N1 genotype in the swine farms in Northern Italy, representing 58.3% of all the H1N1 subtypes isolated [29].

The 2021 HA-1A.3.3.2 strain belonged to a new genetic cluster of SwIAVs recently reported in the Italian domestic swine population (sub-cluster *a*—Figure 3), which is provisionally named genotype 31 [29]. These viruses are reassortants with different combinations of internal genes (pdm09 and avian-origin) (Table 2), which are antigenically different from the previously identified Italian HA-1A strains and have recently been detected in several pig farms in Northern Italy since 2020 [29].

This study represents an ongoing evaluation of influenza A viral circulation in the Emilia-Romagna wild boar population, despite some results limitations. It represents an assessment of SwIAV dynamics in wild hosts, even though the low percentage of subtyped samples as well as isolated strains affected the evaluation of the viral genotypic diversity. The NGS analysis and the genotyping results highlight the simultaneous circulation of the same viral genotypes in overlapping years in both pigs and wild boars; these results support the hypothesis of spillover events between the domestic and the wild reservoirs, with the continuous introduction of SwIAV viral strains at the wildlife–livestock interface.

Furthermore, the demonstration of the continuous introduction of domestic SwIAVs into the Emilia-Romagna wild boar population represents a biosecurity problem in this geographical area, which has a high density of commercial swine farms. Italian farms’ biosecurity needs to be improved and constantly monitored in order to prevent disease transmission via the wildlife–livestock interface, also considering the recent introduction of African swine fever (ASF) in wild boars in the Piedmont region and the Rome province [43,44].

## 5. Conclusions

This study confirmed SwIAV viral circulation in wild boars and represents an upgrade on the research into the Italian situation. Considering the *Sus scrofa* species’ role in the development of reassortant strains, which are agents of spillover infections, and livestock’s potential role as an epidemiological bridge between wildlife and humans [1,2], the dynamic control of the viral hypervariability through systematic surveillance against SwIAVs in the wild boar population is certainly needed.

## Figures and Tables

**Table 1:** SwIAV-positive samples subtyped during the study.

Date	Laboratory Identification	Sampling Province	Subtype
9 January 2018	2018/7179	Reggio Emilia	Not typed
29 December 2018	2019/3529	Ravenna	H1avN1 (clade HA-1C)
8 January 2019	2019/7109	Ravenna	Not typed
22 January 2019	2019/25042	Ravenna	Not typed
15 November 2019	2019/395623	Ravenna	Not typed
28 January 2020	2020/32567	Ravenna	Not typed
8 July 2020	2020/200093	Parma	H1avNx (clade HA-1C)
27 July 2020	2020/223686	Parma	Not typed
30 July 2020	2020/227865	Parma	H1avN1 (clade HA-1C)
30 November 2020	2020/390371	Forlì-Cesena	Not typed
1 December 2020	2020/392593-3	Bologna	H1pdm09N1 (clade HA-1A)
1 December 2020	2020/392593-4	Bologna	H1N2 (clade HA-1B)
10 December 2020	2020/403848	Forlì-Cesena	Not typed
22 December 2020	2020/421894	Forlì-Cesena	Not typed
4 January 2021	2021/214	Forlì-Cesena	Not typed
26 January 2021	2021/27695	Forlì-Cesena	H1pdm09N1 (clade HA-1A)
26 January 2021	2021/27709	Forlì-Cesena	H1pdm09N1 (clade HA-1A)

**Table 2:** Data analysis of the genotypes among the H1N1 subtype detected in wild boars; strains were assigned to the corresponding genotype, as previously described [13,15,37]. The wild boars’ SwIAVs were compared to the strains circulating in swine farms (data from [29]).

Subtype	Nomenclature	HA	NA	PB2	PB1	PA	NP	M	NS	%	Wild Boars’ ID
H1N1	A	1C.2.1	av	av	av	av	av	av	av	58.3%	
		1C.2.1	av	av	av	av	av	av	av	-	**2020/227865**
		1C.2	av	av	av	av	av	av	av	19.3%	
		1C.2.2	av	av	av	av	av	av	av	4.3%	
	M	1C.2.1	av	av	av	av	av	pdm	av	0.5%	
		1C.2	av	av	av	av	av	pdm	av	1.6%	
	U	1C.2.1	av	pdm	pdm	pdm	pdm	pdm	pdm	1.1%	
		1C.2.1	av	pdm	pdm	pdm	pdm	pdm	pdm	-	**2019/3529**
	P	1A.3.3.2	pdm	pdm	pdm	pdm	pdm	pdm	pdm	8.6%	
	31	1A.3.3.2	av	pdm	pdm	pdm	pdm	pdm	av	4.3%	
		1A.3.3.2	av	pdm	pdm	pdm	pdm	pdm	av	-	**2021/27695**
	32	1A.3.3.2	av	pdm	pdm	pdm	pdm	pdm	pdm	1.6%	
	H	1B.1.2.2	av	av	av	av	av	av	av	0.5%	

## Data Availability

The datasets supporting the conclusions of this study are included in the article and in the Supplementary Files. The sequences of the strains of this study are openly available in the GenBank database (https://www.ncbi.nlm.nih.gov/genbank/, accessed on 29 April 2022).

## Supplementary Materials

The following supporting information can be downloaded at: https://www.mdpi.com/article/10.3390/ani12121593/s1. Supplementary File S1: dataset of sequences used in this study.
