# Supplementary material for: Influenza A in Wild Boars: Viral Circulation in the Emilia-Romagna Region (Northern Italy) between 2017 and 2022
Source: Animals (Basel). 2022 Jun 20;12(12):1593. doi: 10.3390/ani12121593 (PMC9220169; doi:10.3390/ani12121593)
Supplement: Supplementary file 1 [file animals-12-01593-s001.zip › animals-1751452-supplementary.pdf]

A/swine/Italy/35129/2019

A/swine/Italy/632/2017

A/swine/Italy/124025/2019

A/swine/Italy/620/2019

A/swine/Italy/172622/2017

A/swine/Italy/18-170725/2018

A/swine/Italy/60591/2018

A/swine/Italy/438757/2019

A/swine/Italy/53693/2020

A/swine/Italy/150383-1/2014

A/swine/Italy/327497-7/2014

A/swine/Italy/196010/2017

A/swine/Italy/74357/2017

A/swine/Italy/300976/2020

A/swine/Italy/81196/2018

A/swine/Italy/10041/2018

A/swine/Italy/220402/2018

A/swine/Italy/366402/2018

A/swine/Italy/327872-86/2020

A/swine/Italy/217458-1/2020

A/swine/Italy/82784/2018

A/swine/Italy/111350/2018

A/swine/Italy/145488/2020

A/swine/Italy/319759/2020

A/swine/Italy/294638/2020

A/swine/Italy/65951/2019

A/swine/Italy/365672/2020

A/swine/Italy/365702/2020

A/swine/Italy/373090/2019

A/Swine/Italy/248971/2019

A/swine/Italy/161875/2019

A/swine/Italy/417841/2019

A/swine/Italy/185280/2020

A/swine/Italy/338367/2020

A/swine/Italy/308263/2020

A/swine/Italy/283256/2020

A/swine/Italy/57984/2019

A/swine/Italy/326711/2018

A/swine/Italy/202268/2019

A/swine/Italy/134212/2019

A/swine/Italy/61121/2018

A/swine/Italy/314232/2018

A/swine/Italy/118860/2019

A/swine/Italy/126300/2019

A/swine/Italy/410927/2018

A/swine/Italy/335210/2018

A/swine/Italy/334694-1/2014

A/swine/Italy/293841/2013

A/swine/Italy/315977/2011

A/swine/Italy/271152/2014

A/swine/Italy/154035/2019

A/swine/Italy/373297/2020

A/swine/Italy/20-113978/14/2020

A/swine/Italy/259446/2020

A/swine/Italy/20-30224/4/2020

A/swine/Italy/361741/2019

A/swine/Italy/222905/2019

A/swine/Italy/98256/2018

A/swine/Italy/115584/2018

A/swine/Italy/83983/2018

A/swine/Italy/15878/2019

A/swine/Italy/351300/2020

A/swine/Italy/361695/2019

A/swine/Italy/353864/2019

A/swine/Italy/270892/2018

A/swine/Italy/389633/2018

A/swine/Italy/312575/2019

A/swine/Italy/202244/2019

A/swine/Italy/389736/2018

A/swine/Italy/370826/2018

A/swine/Italy/386135/2018

A/swine/Italy/386145/2018

A/swine/Italy/161708/2018

A/swine/Italy/170610/2018

A/swine/Italy/58269/2018

A/swine/Italy/24568/2017

A/swine/Italy/1932/2017

A/swine/Italy/89644/2017

A/swine/Italy/37450/2017

A/swine/Italy/104730/2018

A/swine/Italy/366605/2018

A/swine/Italy/299471/2018

A/swine/Italy/47471/2020

A/swine/Italy/85218/2010

A/swine/Italy/198930-3/2013

A/swine/Italy/186822/2011

A/swine/Italy/229537/2011

A/swine/Italy/321986/2009

A/swine/Italy/289700/2009

A/swine/Italy/166015/2010

A/swine/Italy/118616/2010

A/swine/Italy/76687/2010

A/swine/Italy/16959/2011

A/swine/Italy/170177/2010

A/swine/Italy/275736/2010

A/swine/Italy/38272/2010

A/swine/Italy/149992/2010

A/swine/Italy/320546/2009

A/swine/Italy/81062/2009

A/swine/Italy/81226/2009

A/swine/Italy/70757/2009

A/swine/Italy/274298/2009

A/swine/Italy/59209/2009

A/swine/Italy/254261/2010

A/swine/Italy/134110/2011

A/swine/Italy/282964/2010

A/swine/Italy/328356/2014

A/swine/Italy/337085/2012

A/swine/Italy/214856/2017

A/swine/Italy/27719/2017

A/swine/Italy/95312/2017

A/swine/Italy/41350/2011

A/swine/Italy/34333/2014

A/swine/Italy/125010-1/2014

A/swine/Italy/114922/2014

A/swine/Italy/352331/2014

A/swine/Italy/165246/2014

A/swine/Italy/36662-2/2014

A/swine/Italy/346150/2013

A/swine/Italy/91602/2014

A/swine/Italy/3592/1999

A/swine/Italy/172336/2001

A/swine/Italy/1081/00

A/swine/Italy/57669/2011

A/swine/Italy/57680/2011

A/swine/Italy/308725/2011

A/swine/Italy/262270/2018

A/swine/Italy/166277-2/2014

A/swine/Italy/60823/2010

A/swine/Italy/329173/2018

A/swine/Italy/11275/2017

A/swine/Italy/70445/2017

A/swine/Italy/4675/2003

A/swine/Italy/191985/2009

A/swine/Italy/20333/2006

A/swine/Italy/356019/2020

A/swine/Italy/384788/2020

A/swine/Italy/404590/2020

A/swine/Italy/391086/2020

A/swine/Italy/408134/2020

A/swine/Italy/400458/2020

A/wildboar/Italy/27695/2021

A/swine/Italy/303027/2020

A/swine/Italy/74920/2020

A/swine/Italy/326443/2020

A/swine/Italy/187080/2020

A/swine/Italy/376085/2020

A/swine/Italy/377432/2020

A/swine/Italy/105389/2014

A/swine/Italy/212158-2/2020

A/swine/Italy/17-24801-21/2017

A/swine/Italy/18-82500/2018

A/swine/Italy/19-61749-11/2019

A/swine/Italy/321118-6/2014

A/swine/Italy/19-23998-3/2019

A/swine/Italy/361699/2019

A/swine/Italy/20-17132-10/2020

A/swine/Italy/297520/2018

A/swine/Italy/76704/2019

A/swine/Italy/18-57405-3/2018

A/swine/Italy/18/64849-32/2018

A/swine/Italy/18-109888-31/2018

A/swine/Italy/18-82770-28/2018

A/swine/Italy/124953/2014

A/swine/Italy/246087/2014

A/swine/Italy/406037/2019

A/swine/Italy/363738/2019

A/swine/Italy/81938/2019

A/Italy/7838/2019

A/Italy/9368/2019

A/swine/Italy/18-45794-37/2018

A/swine/Italy/60023/2019

A/swine/Italy/26034/2019

A/swine/Italy/179057/2015

A/swine/Italy/6352-17/2013

A/swine/Italy/278299/3/2012

A/swine/Italy/153511-11/2013

A/swine/Italy/287785/2012

A/swine/Italy/224790-2/2012

A/swine/Italy/278299/2/2012

A/swine/Italy/73449/2013

A/swine/Italy/282866/2013

A/swine/Italy/290271/2009

A/swine/Italy/116114/2010

A/swine/Italy/85429/2009

A/swine/Italy/85437/2009

A/swine/Italy/325451/2011

A/swine/Italy/225349-1/2012

A/swine/Italy/225349-2/2012

A/swine/Italy/225349-4/2012

A/swine/Italy/120336/2012

A/swine/Italy/129711-7/2020

A/swine/Italy/97771/2020

A/swine/Italy/138079/2018

A/swine/Italy/117509/2018

A/swine/Italy/47694/2018

A/swine/Italy/147877/2018

A/swine/Italy/63691/2018

A/swine/Italy/280615/2019

A/swine/Italy/210431/2018

A/swine/Italy/245199/2018

A/swine/Italy/37307/2020

A/swine/Italy/332196/2019

A/swine/Italy/391175/2019

A/swine/Italy/85767/2019

A/swine/Italy/49456/2019

A/swine/Italy/334383/2018

A/swine/Italy/372062/2018

A/swine/Italy/19-162065-4/2019

A/swine/Italy/13-163032/2013

A/swine/Italy/60011-3/2014

A/swine/Italy/19-142834/2019

A/swine/Italy/19-142834-30/2019

A/swine/Italy/18-45794-40/2018

A/swine/Italy/75997/2020

A/swine/Italy/288477/2018

A/swine/Italy/20-18057-6/2020

A/swine/Italy/19-66854/2/2019

A/swine/Italy/78247/2020

A/swine/Italy/78383/2020

A/swine/Italy/348542/2018

A/swine/Italy/359045/2019

A/swine/Italy/45510/2019

A/swine/Italy/53981/2019

A/swine/Italy/115494/2019

A/swine/Italy/366771/2017

A/swine/Italy/290807/2017

A/swine/Italy/354072/2017

A/swine/Italy/61157/2018

A/swine/Italy/410546/2018

A/swine/Italy/362647/2019

A/swine/Italy/63033/2020

A/swine/Italy/320530/2019

A/swine/Italy/324970/2019

A/swine/Italy/426372/2019

A/swine/Italy/384889/2018

A/swine/Italy/127069/2020

A/swine/Italy/149878/2013

A/swine/Italy/132401-2/2014

A/swine/Italy/86554/2020

A/swine/Italy/282881/2013

A/swine/Italy/349492/2013

A/swine/Italy/303612/2011

A/swine/Italy/251972/2019

A/swine/Italy/50634/2019

A/swine/Italy/317571/2018

A/swine/Italy/318091/2018

A/swine/Italy/38193/2019

A/swine/Italy/50693/2019

A/swine/Italy/17-55088-15/2017

A/swine/Italy/357504/2017

A/swine/Italy/107012/2017

A/swine/Italy/58197/2015

A/swine/Italy/17-55088-13/2017

A/swine/Italy/58769/2010

A/swine/Italy/20-69081/12/2020

A/swine/Italy/19-162921-14/2019

A/swine/Italy/19-163539-16/2019

A/swine/Italy/381442/2020

A/swine/Italy/400367/2020

A/swine/Italy/127010/2018

A/swine/Italy/419722/2019

A/swine/Italy/85074/2020

A/swine/Italy/141142-2020

A/swine/Italy/332220/2020

A/swine/Italy/326417/2020

A/swine/Italy/20-98963/3/2020

A/swine/Italy/119396/2020

A/swine/Italy/120718/2020

A/swine/Italy/258481/2020

A/swine/Italy/331742/2018

A/swine/Italy/24047/2019

A/swine/Italy/69273/2020

A/swine/Italy/94296/2020

A/swine/Italy/340406/2020

A/swine/Italy/366511/2018

A/swine/Italy/361735/2019

A/swine/Italy/19-125153/7/2019

A/swine/Italy/19-125153/19/2019

A/swine/Italy/282098/2018

A/swine/Italy/382158/2018

A/swine/Italy/343030/2018

A/swine/Italy/406588/2018

A/swine/Italy/22131/2018

A/swine/Italy/169335/2018

A/swine/Italy/87619/2018

A/swine/Italy/330785/2017

A/swine/Italy/301275/2017

A/swine/Italy/VI147/1981

A/swine/Italy/147/1981

A/swine/Italy/1498/2/97

A/swine/Italy/1513-1/1998

A/swine/Italy/101797/2012

A/swine/Italy/17-137733-46/2017

A/swine/Italy/17-66778-1/2017

A/swine/Italy/17-147155-17/2017

A/swine/Italy/17-147155-16/2017

A/swine/Italy/17-165437/2017

A/swine/Italy/17-2895/2017

A/swine/Italy/234480/2017

A/swine/Italy/236825/2019.2

A/swine/Italy/364184/2019.2

A/swine/Italy/17-34594-3/2017

A/swine/Italy/228721/2017

A/swine/Italy/30190/2020

A/swine/Italy/31079/2019

A/swine/Italy/18374/2020

A/swine/Italy/89304/2020

A/swine/Italy/445719/2019

A/swine/Italy/286630/2019

A/swine/Italy/349026/2019

A/swine/Italy/339060/2018

A/swine/Italy/102227/2019

A/swine/Italy/27220/2019

A/swine/Italy/50941/2020

A/swine/Italy/373197/2019

A/swine/Italy/18-170704/2018

A/swine/Italy/14348/2019

A/swine/Italy/165548/2018

A/swine/Italy/351722/2020

A/swine/Italy/352075/2020

A/swine/Italy/31085/2019

A/swine/Italy/190433/2019

A/swine/Italy/34051/2019

A/swine/Italy/34135/2019

A/swine/Italy/351544/2017

A/swine/Italy/349779/2017

A/swine/Italy/265041/2019

A/swine/Italy/327872-36/2020

A/swine/Italy/181296/2019

A/swine/Italy/372968/2019

A/swine/Italy/267654/2018

A/swine/Italy/321022/2018

A/swine/Italy/367671/2019

A/swine/Italy/185838/2019

A/swine/Italy/332816/2019

A/swine/Italy/400733/2019

A/swine/Italy/428424/2019

A/swine/Italy/432243/2019

A/swine/Italy/347423/2020

A/swine/Italy/38652/2019

A/swine/Italy/39576-2/2019

A/swine/Italy/39576-3/2019

A/swine/Italy/294170/2019

A/swine/Italy/313439/2018

A/swine/Italy/314316/2020

A/swine/Italy/215174/2018

A/swine/Italy/216576/2018

A/swine/Italy/143190/2017

A/swine/Italy/44482/2017

A/swine/Italy/344226/2017

A/swine/Italy/404229/2018

A/swine/Italy/202284/2019

A/wildboar/Italy/227865/2020

A/swine/Italy/157306/2018

A/swine/Italy/58902/2018

A/swine/Italy/75555/2018

A/swine/Italy/82829/2018

A/swine/Italy/70101/2018

A/swine/Italy/236197/2018

A/swine/Italy/236808/2018

A/swine/Italy/3741/2019

A/swine/Italy/104881/2018

A/swine/Italy/89579/2019

A/swine/Italy/76982/2018

A/swine/Italy/396630/2019

A/swine/Italy/353418/2018

A/swine/Italy/40277/2019

A/swine/Italy/366609/2018

A/swine/Italy/371405/2018

A/swine/Italy/198140/2019

A/swine/Italy/406597/2018

A/swine/Italy/259437/2018

A/swine/Italy/328966/2018

A/swine/Italy/267012/2017

A/swine/Italy/360158/2017

A/swine/Italy/323257/2017

A/swine/Italy/252160/2018

A/swine/Italy/365724/2017

A/swine/Italy/91162/2018

A/swine/Italy/90903/2018

A/swine/Italy/332188/2019

A/swine/Italy/371231/2019

A/swine/Italy/101364/2017

A/swine/Italy/290024/2017

A/swine/Italy/273623/2018

A/swine/Italy/273034/2018

A/swine/Italy/19-176304-1/2019

A/swine/Italy/273910/2018

A/swine/Italy/19-176305/7/2019

A/swine/Italy/20-5703-2/2020

A/swine/Italy/20-5709-1/2020

A/swine/Italy/20-5707-1/2020

A/swine/Italy/37481/2020

A/swine/Italy/196544/2017

A/swine/Italy/200837/2017

A/swine/Italy/57897/2018

A/swine/Italy/34288/2018

A/swine/Italy/366549/2018

A/swine/Italy/366456/2018

A/swine/Italy/276137/2018

A/swine/Italy/61145/2018

A/swine/Italy/256843/2018

A/swine/Italy/124425/2017

A/swine/Italy/112082/2017

A/swine/Italy/116741/2017

A/swine/Italy/72492/2017

A/swine/Italy/308892/2017

A/swine/Italy/179808/2017

A/swine/Italy/314825/2018

A/swine/Italy/88629/2017

A/swine/Italy/308740/2018

A/swine/Italy/101803/2018

A/swine/Italy/122567/2012

A/swine/Italy/327669/2013

A/swine/Italy/400317/2020

A/swine/Italy/11643/2017

A/swine/Italy/58247/2020

A/swine/Italy/224790/1/2012

A/swine/Italy/274551/2011

A/swine/Italy/195369/2010

A/swine/Italy/138746/2014

A/swine/Italy/311368/2013

A/swine/Italy/5033/2017

A/swine/Italy/122595/2012

A/swine/Italy/290104/2012

A/swine/Italy/319102/2010

A/swine/Italy/244784/2014

A/swine/Italy/26654/2012

A/swine/Italy/98113/2017

A/swine/Italy/302247/2011

A/swine/Italy/16235/2013

A/swine/Italy/317814/2019

A/swine/Italy/317815/2019

A/swine/Italy/72502/2017

A/swine/Italy/236307/2019

A/swine/Italy/444914/2019

A/swine/Italy/31684/2020

A/swine/Italy/179794/2014

A/swine/Italy/18-92341-2/2018

A/swine/Italy/87590/2017

A/swine/Italy/7821/2011

A/wildboar/3529-2/2019

A/swine/Italy/19-49885-4/2019

A/swine/Italy/76625/2019

A/swine/Italy/17-66671-3/2017

A/swine/Italy/161887/2018

A/swine/Italy/366232/2017

A/swine/Italy/23341/2017

A/swine/Italy/84796/2017

A/swine/Italy/54484/2017

A/swine/Italy/86451/2017

A/swine/Italy/174315/2017

A/swine/Italy/263752/2014

A/swine/Italy/351054/2018

A/swine/Italy/253451/2017

A/swine/Italy/29086/2019

A/swine/Italy/98240/2017

A/swine/Italy/81220/2017

A/swine/Italy/302073/2014

A/swine/Italy/302661/2014

A/swine/Italy/327666/2013

A/swine/Italy/244504/2013

A/swine/Italy/191002/2013

A/swine/Italy/198930-1/2013

A/swine/Italy/14-30549/2014

A/swine/Italy/133457-3/2014

A/swine/Italy/130452/2013

A/swine/Italy/23/2017

A/swine/Italy/326226/2017

A/swine/Italy/359202/2013

A/swine/Italy/233405/2012

A/swine/Italy/28762-3/2013

A/swine/Italy/24367/2017

A/swine/Italy/224721/2012

A/swine/Italy/280201/2013

A/swine/Italy/329017/2011

A/swine/Italy/51411/2014

A/swine/Italy/302593/2010

A/swine/Italy/218884-2/2012

A/swine/Italy/269814/2013

A/swine/Italy/151672/3/2003

A/swine/Italy/328533/2017

A/swine/Italy/311691/2020

A/swine/Italy/181051/2017

A/swine/Italy/317775/2010

A/swine/Italy/63580/2010

A/swine/Italy/217407/2017

A/swine/Italy/238098/2014

A/swine/Italy/87434/2014

A/swine/Italy/281230-1/2014

A/swine/Italy/333140/2014

A/swine/Italy/107798/2012

A/swine/Italy/229528/2011

A/swine/Italy/267505/2010

A/swine/Italy/195399/2012

A/swine/Italy/131916/2011

A/swine/Italy/128948/2014

A/swine/Italy/280525/2017

A/swine/Italy/319204/2017

A/swine/Italy/319217/2017

A/swine/Italy/55230/2012

A/swine/Italy/50814/2012

A/swine/Italy/96500/2014

A/swine/Italy/50255/2013

A/swine/Italy/304621/2014

A/swine/Italy/310411/2009
